# Notes and News

**Published:** 1902-11-08

**Authors:** 


### NOTES AND NEWS.

At Rangoon a new asylum for female lepers has been opened.

Madam Vassiliades has been appointed physician to the prison for women at Athens.

Mr. Clifford Probyn, Mayor of the City of Westminster, will preside at the festival dinner of the Royal Westminster Ophthalmic Hospital on December 5th at the Hotel Cecil.

Two entertainments in aid of charities are announced to take place before the end of the year. On behalf of the League of Mercy, the lady presidents of the Western Metropolitan districts of Chelsea, Fulham, Hammersmith, Hampstead, Kensington, Marylebone, Paddington, St. George's, Hanover Square, and Westminster, are holding a dance, under the patronage of T.R.H. the Prince and Princess of Wales, at the Empress Rooms, Royal Palace Hotel, Kensington, on December 10th, to further the objects of the League which was founded by Royal Charter to promote the welfare of the Metropolitan hospitals by securing support for King Edward's Hospital Fund. Amongst the lady presidents and presidents who are patronising this effort are the Countess Grosvenor, the Countess Cadogan, the Lady Farquhar, the Duke of Argyll, the Earl of Kilmorey, Lord Farquhar, Lord Wolverton, and Sir Henry Burdett. Mrs. Leopold Rothschild has kindly promised to decorate the ballroom with palms and flowers. Information can be obtained from the Lady Presidents.

In aid of the West Ham Hospital a grand mask and domino ball is to be held on Monday, December 15th, at the Empress Rooms, Royal Palace Hotel, Kensington. The lady patronesses include the Countess of Pembroke, the Countess of Dudley, the Countess of Eglinton, the Countess of Lytton, the Countess of St. Germans, the Countess of Lonsdale, and many other distinguished members of society. Vouchers for the ball will only be issued by the ladies' committee, under the presidency of Lady Maud Wilbraham, and the price of each ticket will be one guinea, which includes supper and champagne.

A new wing has been opened at the Port Sanitary Hospital at Denton, Gravesend.

The Dawlish Cottage Hospital and the Dawlish Infirmary have been amalgamated.

A public dispensary has been opened in connection with the Royal Alexandra Hospital at Paisley.

A dental department has been opened at the West Bromwich Hospital, the cost of which has been defrayed by Sir Ernest Spencer.

Sir Henry Burdett addressed two crowded meetings last week in aid of the League of Mercy, at Croydon and Woolwich respectively.

Mr. George Armitstead has presented £1,000 to King Edward's Hospital Fund, in addition to his contribution of £500 made earlier in the year.

The Birmingham Hospital Saturday Fund again shows an advance in its collection. Last year it amounted to £17,544, and this year it has reached £18,067.

Major Ross will deliver the Harben Lectures this year at King's College on November 10th, 11th, and 12th, at 5 p.m. The subject is Intermittent Fever. The lectures are open to the public.

The prize of £500, offered by the Secretary of State for War, for the best designed ambulance waggon for service during war in rough countries more especially, has been gained by Messrs. Holmes and Co., coachmakers. The ambulance is capacious, carrying four recumbent of 12 sitting patients, with driver and two orderlies. Stretchers and all medical and surgical appliances are provided for.

At a meeting of the Council of King's College, London, Professor William Rose, M.B., F.R.C.S., was elected a member of the Council, and was also elected Emeritus Professor of the College, and Consulting Surgeon to King's College Hospital. Professor Rose, who in 1893 succeeded Lord Lister as Senior Surgeon to King's College Hospital, has been for upwards of 26 years a member of the medical faculty of the College.

Mr. Alderman Treloar once again appeals for subscriptions to the fund for providing a New Year's entertainment to about 1,200 poor children of the Ragged School Union in the Guildhall of the City of London, and the accompanying distribution of Christmas hampers to 4,000 or 5,000 little cripples. No one, he says, who has witnessed the reception by a home-tied cripple of a hamper at Christmas can over-estimate the good done by the timely gift, and the Guildhall banquet is a source of never-failing pleasure. His Majesty the King has contributed to the fund as he has done for several years past. Subscriptions, large or small, to the "Children's Fund" may be sent to Alderman Treloar at 69 Ludgate Hill, E.C.

